# Gender (in)equality in nordic ambulance services: do ambulances have glass ceilings?

**DOI:** 10.1186/s13049-025-01358-7

**Published:** 2025-03-17

**Authors:** Christoffer Ericsson, Veronica Lindström, Jeanette Viggen Andersen, Trine Møgster Jørgensen, Jonas Alex, Anu Venesoja

**Affiliations:** 1 Graduate School and Research, Arcada University of Applied Sciences, Helsinki, Finland; 2 Faculty of Medicine, University of Helsinki, Helsinki, Finland; 3 Department of Nursing, Division of Ambulance Service, Region Västerbotten, Umeå University, Umeå, Sweden; 4 Faculty of Health Sciences, Department for Prehospital Work, Oslo Metropolitan University - OsloMet, Oslo, Norway; 5 Faculty of Health and Social Care, LAB University of Applied Sciences, Lappeenranta, Finland; 6 Department of Emergency Care and Services, University of Helsinki and Helsinki University Hospital, Helsinki, Finland

## Abstract

Political efforts in the Nordic countries aim to promote gender equality. However, an assumption is that patriarchal structures remain embedded in EMS organizations, often leading to a ‘glass ceiling’ effect for women. The Emergency Medical Services (EMS), generally positioned at the intersection of safety authorities and healthcare, operates within environments often shaped by masculine values and norms. Concurrently, the service also connects strongly to compassion, caring and nursing, which have been historically female-dominant professions and working environments. In recent decades, more females have entered the EMS. Despite the growing number of female paramedics, challenges persist, particularly in relation to gender inequality and workplace culture. Females in EMS field continue to face gender stereotypes, which may contribute to inequality. Gender stereotypes, combined with research describing sexual harassment and bias, underscore the need for further discussions and research on the impact of gender on paramedic work environments and career pathways for women working in the service.

## Navigating a male-dominated field

A long-standing belief is that there is no gender gap in the Nordic Emergency Medical Services (EMS) and supportive structures may appear to exist for female paramedics’ career opportunities, but do they? Outside of anecdotal evidence and occasional social media campaigns, gender-based workplace cultures in the context of the EMS in the Nordic countries have not been seriously explored, despite its potential impact on the EMS professionals’ well-being and career retention.

EMS professionals operate in a uniquely demanding environment, facing complex and challenging situations daily. Performance expectations faced by paramedics range from managing their tasks, to being socially accepted into the EMS community and a clarity of their professional role [1, 2]. Although EMS goals are similar worldwide, including early recognition of severe illness and injury and management at the scene to save lives, the education, healthcare systems, system activation, culture, equipment, and information technology differ between EMS globally. Differences can be seen even between the Nordic countries in relation to educational standards and integration of systems, although the EMS and the emergency care framework are somewhat similar [3]. The similarities in the Nordic countries’ EMS can be explained by the fact that healthcare and education are mostly tax-funded [4], and political decisions are based on aspects of equality, with an effort to include the underrepresented gender in all different levels in the society. Despite these efforts to achieve gender equality, there are signs of inequality in the Nordic EMS.

To the best of our knowledge, as clinical practitioners, educators, and researchers, in Nordic countries few females are in EMS leadership positions. Supporting our views, similar findings are reported from the United States [5, 6] and Canada [7]. All the while, there are indications that in some EMS, female paramedics are more than twice as likely to have a university degree, compared to their male contemporaries [7]. Reasons for possible inequality are not clear, but it is known that EMS organizations have traditionally been male-dominated working environments with double dominance, both numerically and normatively [8]. Such numerical and normative dominance in the workplace has a risk of being defined by masculine values and heterosexual norms that shape the workplace culture [9, 10]. Is the workplace culture in the EMS a reason for the ‘glass ceiling’ effect on women?

It has been shown that females in the EMS have challenges with male-dominated culture, gender stereotypes, and social constructs that cause unconscious bias and pressures that prevent them from advancing in the EMS [9, 11]. It has further been speculated that while networking can increase social capital and career progression for males, due to the prevalence of existing male leadership, the same cannot be said for female paramedics [11]. Females have questioned their decision to work in the EMS based on their experiences, and a lack of career advancement is described as contributing to female paramedics’ intention to leave the service [5, 6]. In some EMS contexts, there is also a clear pay gap between genders, with female paramedics receiving, on average, lower pay compared to male paramedics in the same organization [11]. Whether this is also true for Nordic EMS remains unclear. Still, as there are indications that male and female do not have the same career conditions and pay, more knowledge is needed on how and if workplace culture impacts gender equality.

Furthermore, it is known that females in male-dominated working environments experience high frequencies of sexual harassment, and the same phenomenon is also reported in EMS settings [5, 9, 12, 13]. This is further exasperated by a perceived high threshold for female paramedics to report sexual misconduct, in fear of negative outcomes [8]. There are further indications of negatively skewed attitudes toward female and non-stereotypical male paramedics [11]. Assertiveness among female paramedics has been described as ‘bossy behaviour’, while oversight of female team members has also been reported in paramedic team communication [11]. Despite such gender-based harassment and inequalities reported in the EMS, women continue to start working in the EMS at higher proportions [14]. In Finland, Sweden, and Norway, official statistics (www.vipunen.fi, https://www.uka.se/vara-resultat/statistik, [15]) show that a majority of new paramedic students are nowadays females. The culture and gender distribution in the EMS may change as more women study to become paramedics. Supporting females progression and position in EMS may lead to questioning and changing traditional cultural norms and organizational behavioural patterns [8]. Whether an increased number of females in Nordic EMS will lead to changed cultural norms is unclear, as there is a risk that they may become new bearers of the existing culture to adapt to the workplace norms.

## Conclusions

### Future of gender equality in nordic ambulance services

Gender-based workplace culture, including harassment and inequality, has received little serious attention in the context of the EMS in the Nordic countries despite its potential impact on the well-being, work efficiency, job satisfaction, and retention of paramedics working in EMS. Within the service, these inherently traditional values, male-dominated culture, and preconceptions may thus lead to a glass ceiling or a “leaky pipeline effect” for females working there. However, it is still unknown whether the phenomena and findings discussed above are true for the Nordic countries’ EMS, or to what extent they are generalizable across Nordic cultures and geographical borders. Clearly there is a need for more knowledge of how equality, inclusion, gender perspectives and the culture in the EMS manifest and potentially affect women paramedics, other minorities, e.g. sexual, ethnic and religious, as well as care of EMS patients. EMS organizations and educational institutions that educate paramedics should critically examine their own practices to identify possible structures and operating models that cause gender inequality.

## Data Availability

No datasets were generated or analysed during the current study.
